# Druggable Biochemical Pathways and Potential Therapeutic Alternatives to Target Leukemic Stem Cells and Eliminate the Residual Disease in Chronic Myeloid Leukemia

**DOI:** 10.3390/ijms20225616

**Published:** 2019-11-10

**Authors:** Fabien Muselli, Jean-François Peyron, Didier Mary

**Affiliations:** Université Côte d’Azur, Institut National de la Santé et de la Recherche Médicale (Inserm) U1065, Centre Méditerranéen de Médecine Moléculaire, CEDEX 3, 06204 Nice, France; fabien.muselli@univ-cotedazur.fr (F.M.); jean-francois.peyron@univ-cotedazur.fr (J.-F.P.)

**Keywords:** chronic myeloid leukemia, leukemic stem cells, resistance, tyrosine kinase inhibitors, microenvironment, apoptosis, autophagy, metabolism, epigenetic, clinical trials

## Abstract

Chronic Myeloid Leukemia (CML) is a disease arising in stem cells expressing the BCR-ABL oncogenic tyrosine kinase that transforms one Hematopoietic stem/progenitor Cell into a Leukemic Stem Cell (LSC) at the origin of differentiated and proliferating leukemic cells in the bone marrow (BM). CML-LSCs are recognized as being responsible for resistances and relapses that occur despite the advent of BCR-ABL-targeting therapies with Tyrosine Kinase Inhibitors (TKIs). LSCs share a lot of functional properties with Hematopoietic Stem Cells (HSCs) although some phenotypical and functional differences have been described during the last two decades. Subverted mechanisms affecting epigenetic processes, apoptosis, autophagy and more recently metabolism and immunology in the bone marrow microenvironment (BMM) have been reported. The aim of this review is to bring together the modifications and molecular mechanisms that are known to account for TKI resistance in primary CML-LSCs and to focus on the potential solutions that can circumvent these resistances, in particular those that have been, or will be tested in clinical trials.

## 1. Introduction

Chronic Myeloid Leukemia (CML) is a myeloproliferative neoplasm characterized by a chromosomic translocation between chromosomes 9 and 22 at a very early stem/progenitor cell level, with the emergence of the well-known chromosome of Philadelphia. The oncogenic event fuses parts of the gene for the c-ABL tyrosine kinase downstream of the *BCR* gene. This creates the constitutively active BCR-ABL tyrosine kinase, at the root of the disease. BCR-ABL supports initiation and progression of CML through a plethora of signaling pathways [1]. If left untreated, CML rapidly evolves from a chronic phase into a blast crisis with a massive accumulation of myeloid cells in the BM and the blood. This uncontrolled proliferation of Philadelphia positive cells (Ph+) supplants normal hematopoiesis, with a gradual replacement of normal blood cells.

The very first treatments developed with Hydroxyurea, Busulfan or Interferon-Alpha (IFN-α)-based therapies have shown their limitation to affect BCR-ABL proliferative cells and thereby to keep the disease in check [2]. CML was the first cancer to benefit from a targeted therapy in the early 2000s with STI571/Imatinib, a tyrosine kinase inhibitor (TKI), that specifically blocks ABL activity. This treatment dramatically improved the therapeutic outcome of the patients, with 95% of them achieving a complete hematological remission (CHR) [3]. Furthermore, second- (Dasatinib/BMS354825, Nilotinib/AMN107, Bosutinib/SKI-606) and third- (Ponatinib/AP24534) generation TKIs have been designed to bypass primary and secondary resistances to Imatinib [4]. The rise of these TKIs has drastically improved CML patients’ outcome and survival, redefining CML from an incurable disease to a manageable one. While TKIs, especially the second-generation ones, are very efficient to eliminate blasts, they remain nonetheless toxic for healthy cells in the long run with numerous side effects affecting the gastrointestinal tract or the cardiovascular system [5].

A discontinuation of Imatinib has therefore been tested once the disease is undetectable at the molecular level. Unfortunately, half of the patients in this study relapsed within two years [6], supporting the idea of a residual disease sustained by a discrete population of Leukemic Stem Cells (LSCs), that are insensitive to treatments, capable to self-maintain and to reinitiate the disease in the long-term. Therefore, successfully achieving a cure requires the elimination of LSCs. Most of the time, LSCs are in a quiescent state in the bone marrow (BM) and thus insensitive to TKI monotherapy. This is why during the last decade, many research groups have been deciphering the pathways involved in LSC maintenance and expansion, to propose numerous pertinent approaches to eradicate them specifically.

Most dysregulations connected to TKI resistance in CML are exclusively observed on cell lines, but some of them were also found in primary CD34^+^ CML cells.

**Figure 1:**
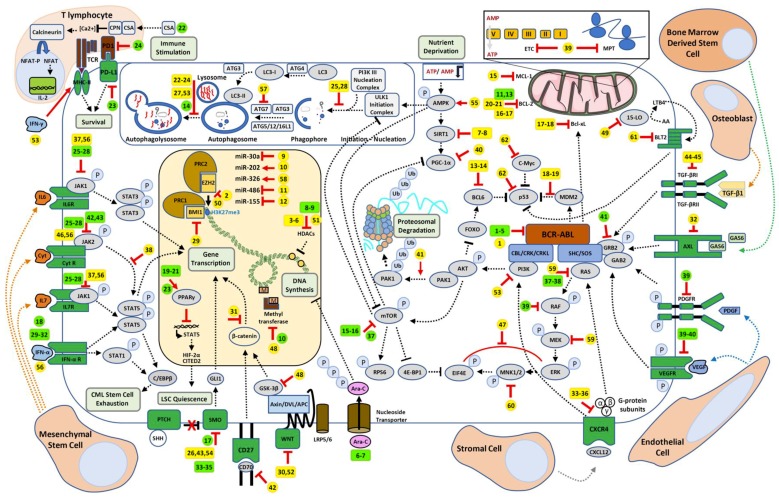
Chronic Myeloid Leukemia (CML) Leukemic Stem Cells (LSC) pathways involved in tyrosine kinase inhibitor (TKI) resistance and potential therapeutic targets to impair them. LSC (in the center) is represented within its microenvironment and key interactions with different bone-marrow cells are shown. This figure is coupled with Table 1 for ex-vivo candidate molecules (yellow tags) and Table 2 for clinical trials involving candidate molecules (green tags) with their respective mode of action (red symbols).

The present review is focused on TKI-resistance processes observed ex-vivo for which pharmacological targeting has been demonstrated to resensitize LSCs to TKIs (Table 1) eventually given rise to clinical trials (Table 2), summarized in a global overview (Figure 1).

## 2. Leukemic Stem Cells: Cannot See the Wood for the Trees

LSCs were first identified in Acute Myeloid Leukemia (AML) within the CD34^+^/CD38^−^ cell population and defined as cells that can engraft and initiate AML in a NOD/SCID immunodeficient host mouse [65]. They have the ability to self-renew and also to produce differentiation-impaired leukemic blasts [65]. LSCs have also been described in other leukemias such as CML [66] or B-lineage acute lymphoblastic leukemia [67], and Cancer Stem Cells (CSCs) have been identified in numerous solid cancers such as breast [68], colorectal [69] or brain [70] cancers, unravelling their crucial importance in cancerology.

LSCs that also referred as Leukemia-Initiating Cells (LICs), represent a very small subset of CML cells, residing in the very complex microenvironment of the BM. Contrary to proliferating leukemic cells, LSCs have been shown to be “BCR-ABL oncogene independent” as demonstrated by their capacity to maintain and reinitiate the disease even after BCR-ABL activity has been shut down in the SCLtTA/BCR-ABL double transgenic CML mouse model [71]. Furthermore, despite a continuous inhibition of BCR-ABL by TKIs (Imatinib, Dasatinib or Nilotinib), primary CML stem/progenitors are able to self-maintain in the presence of a cocktail of cytokines [72]. Since LSCs emerge from HSCs, the two types of cells cannot be formerly distinguished from each other from their phenotype. The direct identification of LSCs and evaluation of their numbers in BM or blood sample is not feasible yet, and the gold standard model to study LSCs remains the serial transplantation in recipient mice, of samples containing LSCs, followed by monitoring the development of leukemia [73].

Many studies have found that specific biochemical dysregulations in LSCs can contribute to their resistance to TKI treatments, resulting in their persistence in the bone marrow microenvironment (BMM). It is currently accepted that bi-therapies combining TKIs with various drugs that target these dysregulations could lead to elimination of LSCs.

## 3. BCR-ABL Dependent and Independent Resistances

### 3.1. BCR-ABL-Dependent Resistances and Alternative Solutions

Emergence of point mutations in BCR-ABL, essentially in the different structural domains of ABL1: Phosphate-binding loop (P-loop), Activation-loop (A-loop), SRC Homology 2/3 Domain (SH2/SH3) and catalytic domains represent the most encountered forms of BCR-ABL-dependent resistances [74]. They are usually the consequence of long-term adaptation of leukemic cells upon TKI treatments. Those mutations are mostly single ones although double mutants have also been identified, with some of them refractory to all TKIs. Some mutations can confer resistance to some or to all TKIs. For instance, the gatekeeper mutations affecting threonine 315: T315I and T315V are only sensitive to Ponatinib, the third generation of TKIs. As the disease progresses towards accelerated or blast crisis phases, genomic instabilities can favor double mutations on BCR-ABL such as T315I/G250E, T315I/Q252H, T315I/Y253H, T315I/E255V, T315I/F311, T315I/M351T, T315I/F359V, T315I/H396R or T315I/E453K which are associated with poor prognosis as most of them generate insensitivity to all available TKIs [75].

Imatinib, the first synthetized TKI, targets the ATP binding site of the AB1 catalytic domain in a type-II-dependent fashion, stabilizing the catalytically inactive conformation of the kinase to induce apoptosis of CML cells. The emergence of 2nd generation TKI was a necessity to treat first-line-Imatinib refractory patients and Dasatinib, Nilotinib and Bosutinib were developed to solve this issue. Dasatinib is a type-I kinase inhibitor (only affecting the catalytically active conformation) while the two other TKIs are type-II kinase inhibitors like Imatinib. In an attempt to treat BCR-ABL T315I refractory patients, Ponatinib was developed to target the active conformation of T315I BCR-ABL using a structure- design strategy. However, some BCR-ABL double mutants still appeared resistant to Ponatinib [75]. Asciminib is the last developed TKI and also the first allosteric BCR-ABL inhibitor that is effective against any mutation in the ATP binding site [76]. Furthermore, combination of Asciminib with Ponatinib is highly efficient against BCR-ABL compound mutants [7]. Indeed, this molecule is currently in five recruiting clinical trials (NCT03906292, NCT03106779, NCT03595917, NCT03578367 and NCT02081378).

In addition to *BCR-ABL* point mutations, a higher expression of BCR-ABL can induce TKI resistance as observed for CD34^+^/BCR-ABL^HIGH^ expressing cells [77]. In the same way, the genomic instability that goes with CML progression towards late phases further increases the occurrence of BCR-ABL mutations. Furthermore, BCR-ABL is known to trigger DNA damages (double-strand breaks) via reactive oxygen species (ROS) stimulation [78] linked to PI3K/mTOR activation [79], which further increases mutagenesis by promoting the emergence of additional mutations.

### 3.2. BCR-ABL-Independent Resistances

Targeting DNA synthesis with the anti-metabolite cytarabine (NCT00022490, NCT00015834) has been first considered as a broad approach to counteract BCR-ABL-independent resistances in CML. During the last two decades, the description at a molecular level of diverse BCR-ABL-independent resistance mechanisms, led to the identification of dysregulated signaling pathways in LSCs. Those dysregulations have paved the way for precise pharmacological interventions to resensitize resistant CML cells to TKIs, even in the case of the T315I “hell” mutation. Several examples are presented below with a specific focus on mechanisms allowing the maintenance of CML-LSCs, and for most cases, on potential therapeutic molecules to target them.

#### 3.2.1. Drug Transporters

LSCs resistance to chemotherapies or TKIs can be partly explained by the high expression of different members of the ATP-binding cassette (ABC) family of transporters able to export out of cancer cells a wide range of molecules, thereby decreasing their therapeutic potential. The high expression of ABCB1 (MDR1, P-glycoprotein), ABCC1 (multidrug resistance protein, MRP1) and ABCG2 (breast cancer resistance protein; BCRP) transporters confers multidrug resistance (MDR) to LSCs. For example, ABCG2 has been shown to efflux Imatinib from cells [80] and to control its delivery in-vivo [81]. Efflux-mediated resistance was shown not to be specific to Imatinib but also to apply to other TKIs [82].

#### 3.2.2. Targeting Epigenetic Dysregulation in CML

Epigenetic modifications at the chromatin control gene expression during proliferation and differentiation of stem cells. Interestingly, an abundant literature reports that the epigenetic signature of cancer stem cells is quite different from their normal counterpart highlighting that an altered epigenetic program may play an important role in cancer progression.

Because numerous epigenetic mechanisms have been reported to be altered during CML progression [83,84] or reprogrammed after TKI therapy [85], their modulation has to be considered in order to counteract with resistance as a means of eradicating residual disease [86].

Proteins of the polycomb family that are part of the Polycomb Repressive Complexes 1 and 2 (PRC1/PRC2) function as repressors of gene expression via chromatin modifications [87]. These two complexes have been involved in the progression of both solid tumors and hematological malignancies [88]. Their epigenetic regulatory function can be affected by alterations in the genes coding for the different components of each complex or through regulation of associated protein partners.

BMI1 a polycomb protein, part of the PRC1 complex, plays an important role in supporting self-renewal of both HSC and LSCs [89]. While there is no clear demonstration of the involvement of BMI1 in CML initiation, the polycomb clearly cooperates with BCR-ABL when introduced in CD34^+^ CML cells to stimulate their proliferation and self-renewal [90]. Furthermore, BMI1 was shown to be overexpressed in CML vs control subjects [91] with an increased expression that mirrors disease progression [91,92] and could thus represent a molecular marker for CML prognosis prediction [93] Moreover, BMI1 could be a potential target. Indeed, CD34^+^ CML cells treated with the BMI1 inhibitor PTC-209, failed to achieve clonogenicity [92] leaving hope for a possible targeting of LSCs.

EZH2, another important polycomb protein, member of the PRC2 complex, was also described as being implicated in CML-LSCs maintenance after mutation or overexpression that triggered an epigenetic reprogramming. Targeting EZH2 by CRISPR/Cas9-mediated gene editing [52] is an efficient strategy to target LICs, and the specific inhibitor GSK343 [8] sensitizes LSCs to TKIs.

ASXL1 that associates with PRC2 is well known to regulate epigenetic marks and transcription. The *ASXL1* gene is frequently mutated in CML diagnosed-patients and often correlated with poor prognosis [94,95], suggesting a role in the progression of the disease [96,97] and could thus represent a future attractive target.

The Histone deacetylase (HDAC) family represents another class of epigenetic regulators that have been extensively described for their implication in cancer biogenesis and in cancer stem cells maintenance. In the case of CML, the first HDAC inhibitor used with success in combination with a TKI to target LSCs is the class I and II dual-HDAC inhibitor SAHA/Vorinostat (Suberoylanilide hydroxamic acid) that was found to enhance Imatinib-induced apoptosis of CD34^+^ CML cells [98]. Other HDAC inhibitors like LBH589 can be combined with Imatinib [83], Nilotinib [10] or LAQ824 to eliminate Imatinib-refractory primary CML cells [83,99]. Furthermore, LBH589 and LAQ824 combined with a TKI gave interesting results in Imatinib-resistant CML cells harboring the BCR-ABL T315I mutation [99,100]. The class I, II and IV HDAC inhibitor Pracinostat, corrects pre-mRNA splicing of the BIM pro-apoptotic protein to overcome BIM deletion polymorphism-induced TKI resistance observed in certain CML cases [53]. Preclinical *in-vitro* studies have shown that the combination of Vorinostat with Dasatinib leads to the death of CML primary cells and can also attenuate the levels of expression of E255K and T315I-BCR-ABL mutants [10]. Combination of HDAC inhibitors (Hydralazine and Magnesium Valproate) with Imatinib gave promising clinical results on Imatinib non-responders [12], and several TKIs/HDAC inhibitors combinations such as Imatinib and Panobinostat (pan HDAC inhibitor): NCT00686218; or Dasatinib and Vorinostat: NCT00816283 were evaluated in clinical trials.

Among the SIRT group that is part of the HDAC family, SIRT1, a NAD-dependent deacetylase has been described, first to be overexpressed in human CML-LSCs [13] and second, to play a role in the regulation of both HSCs and LSCs [101,102]. Furthermore, Wang et al. described that histone deacetylation by SIRT1 was implicated in the acquisition of genetic mutations in *BCR-ABL* associated with TKI resistance and that targeting SIRT1 can be a good strategy to overcome these drug resistances [101]. The specific inhibition of SIRT1 by Tenovin-6 [13] or TV39OH [14] enhances p53 anti-cancer functions via increased acetylation and therefore combination with Imatinib or Nilotinib respectively leads to LSCs targeting.

Activity of promoters can be regulated by methylation-demethylation processes. The role of methylation in CML was already suspected in the pre-TKI era when silencing of cadherin 13, a mediator of calcium-dependent cell-cell adhesion, was demonstrated to correlate with response toIFN-α [103]. Besides, methylation can serve as a prognostic tool. Indeed, the methylation status of the BIM promoter, is associated with an unfavorable prognosis in CML diagnosed patients [104], while an increased BCR promoter methylation correlates with a more favorable response to Imatinib [105].

Otherwise, methylation of some gene promoters was reported to be implicated in CML progression like the one for SOCS1 that stimulates the JAK/STAT activity [29,106] or that of the ATP-dependent RNA helicase DDX43 [107]. Progression of the disease was also demonstrated to correlate with methylation of the promoter for genes implicated in cell-cycle progression (*p15* and *p16*) [108,109,110], apoptosis (*p14 ARF*) [109,110], *p53* [110] and *DAPK* [110,111]. TKI resistance has been associated with methylation of several promoters for *sFRP1* in the Wnt pathway [112], or of the Src suppressor gene *PDLIM4* [113], and of *HOXA4*. Studies in CD34^+^ progenitor cells indicate that methylation particularly concerns tumor suppressor genes and occurs very early during CML transformation before being markedly decreased in the last phase of the disease. More recently, another study examined the DNA methylation profile of CD34^+^/CD15^−^ in early CP-CML patients’ cells and described 18 genes that could be aberrantly repressed upon hypermethylation and 81 upon hypomethylation [114]. This suggests that demethylating agents could be more effective in the first phase of the disease [115]. Interestingly, the PRMT5 methyltransferase, overexpressed in CML-LSCs, can be targeted with the small-molecule inhibitor PJ-68, abrogating the Wnt/β-catenin pathway and leading to the elimination of LSCs [54].

Most of the molecules that modify promoter methylation have only been tested alone. Nevertheless, a phase II study that combined decitabine with Imatinib on a 28-patient CML cohort, showed an improvement in the survival for non-responder patients in advanced phase CML without BCR-ABL kinase mutation [116].

The non-coding RNAs of the long non-coding (lncRNAs) or the miRNAs families represent a recently discovered epigenetic process regulating mRNA levels and protein translation via different mechanisms [117].

Today, only few reports have emerged concerning dysregulations of lncRNAs in CML, although none have been described yet for LSCs. Nevertheless, there is some hope for the development of new therapeutic weapons in the near future. For instance, the lncRNA-HULK which is aberrantly expressed in CML, positively correlates with clinical evolution of the disease, and its down-regulation is sufficient to trigger apoptosis of leukemic cells by repression of c-MYC and BCL-2 [118]. The lncRNA-PLIN2 also upregulated in CML patients by a CEBPA induced-mechanism, increased in turn the expression of AKT, p-AKT, GSK-3β, β-catenin and Axin2/conductin, and could promote tumor growth by activating GSK3 and Wnt/β-catenin signaling in-vivo [119]. The expression level of lncRNA-BGL3 strongly increases following TKI treatment because of a BCR-ABL-dependent repression mechanism involving a c-MYC-dependent methylation [120]. Through its transcriptional interference activity, the lncRNA-MEG3 can regulate miR-21 expression, influencing the levels of MMP-2, MMP-9, BCL-2 and BAX and regulating proliferation and apoptosis [121].

The dysregulation of some miRNAs expression has been frequently reported in CML patients, in the last decade [122,123,124,125], presenting them as potential biomarkers [126] and opening new therapeutic options [86]. A group of 19 miRNAs was identified to predict clinical resistance to Imatinib in newly diagnosed CML [127] and more recently, 8 dysregulated miRNAs were described to be involved in the BCR-ABL-independent resistance to TKI in CML [128]. Moreover, targeting miR30a decreases autophagy and therefore sensitizes primary LMC cells to Imatinib [15], while overexpression of miR-203 overcomes TKI-resistance *in-vitro* [129]. Furthermore, overexpression of miR-202 inhibits hexokinase 2 (HK2) and glycolysis which resensitizes Imatinib-resistant CML cells [16]. Also, inhibition of miR-486, overexpressed in CML progenitors interfered with their growth and survival and sensitized them to Imatinib [17]. The inhibition of BCR-ABL by TKIs is also responsible for increased endogenous miR-126 levels, favoring LSCs quiescence and persistence [130]. Recently, a study showed that combining Imatinib with Ovatodiolide, a natural diterpenoid that up-regulates miR-155 to inhibit PI3K/mTOR, efficiently induced cell death of CD34^+^/CD38^−^ CML cells. [18].

#### 3.2.3. Apoptosis Defects in Resistance

Numerous genes implicated in apoptotic induction were found to be repressed or mutated in CML cells such as the tumor suppressor gene *p53* [131], the pro-apoptotic members *BIM* [104] and *BAD* [132] or members of the anti-apoptotic *BCL2* family [132,133,134,135] like *BCL2*, *Bcl-xL* and *MCL1*. BCR-ABL is a potent cell death inhibitor and was demonstrated to promote CML progression by preventing formation of the caspase-9 apoptosome [136].

Targeting the BCL6/p53 axis could be one option, as BCL6 represses p53. This was achieved on primary human CD34^+^/CD38^−^ CML stem cells using a peptide inhibitor of BCL6 (RI-BPI) [19]. TKI resistance was also described to be associated with a STAT1-dependent upregulation of BCL6 in CD34^+^ CML stem cells and a combination of TKIs with inhibitors of BCL6 (FX1) or MCL1 (A-1210477) is sufficient to overcome this resistance in LSCs [20].

During the last decade, many studies have addressed the potential of targeting BCL2 family members in combination with one TKI, producing convincing results. The pan-BCL2 inhibitor Sabutoclax, associated with BCR-ABL inhibition with Dasatinib, rendered BM-resident blast crisis LSCs, sensitive to the TKI, suggesting a possible elimination of dormant LSCs [21]. Similarly, the BCL2/Bcl-xL inhibitor ABT-737 associated with Imatinib demonstrated strong synergism to induce cell death of both proliferating and quiescent CD34^+^/CD38^−^ TKI-insensitive CML cell populations [22,23]. In two other studies, a cell death response was triggered on primary CML CD34^+^ quiescent cells by a triple combination associating ABT-737 to block BCL2 and Bcl-xL, Nilotinib to inhibit BCR-ABL and a MDM2 inhibitor such as Nutlin3a [24] or DS-5272 [25] to restore p53 activation.

Association of the BH3 mimetic ABT-199 (Venetoclax), a more specific BCL-2 inhibitor, with either Imatinib [26] or Nilotinib [27] synergistically induced death of quiescent stem/progenitor primary CML CD34^+^ cells. Furthermore, the association of Dasatinib with ABT-199/Venetoclax will be evaluated on a phase II clinical trial (NCT02689440) that is recently recruiting. A tri-therapy combining Ponatinib, Venetoclax and Dexamethasone will also be tested in the NCT03576547 trial. Interestingly, Imatinib combined with BCL-2 antisense oligodeoxynucleotide (Oblimersen) was evaluated in a phase II clinical trial (NCT00049192).

#### 3.2.4. Role of Autophagy in CML Resistances

Autophagy enables cells to degrade and recycle their intracellular components in lysosomes, to avoid cell death, being a protective cellular response that occurs under stress conditions and in particular during nutrient depletion [137].

The first evidence of a possible role of autophagy in CML initiation and progression came with the observation that an increased autophagic response occurred in primary CML cells in response to Imatinib and that co-treatment with inhibitors (Chloroquine, Bafilomycin A1) of late stage autophagy, resulted in the elimination of phenotypically and functionally defined CML stem cells [9,28]. Since, numerous studies have demonstrated that chloroquine and other lysosomal inhibitors induce LSCs elimination [39,138]. Furthermore, the combination of hydroxychloroquine with Imatinib is being evaluated in a clinical trial (NCT01227135).

It was then reported that autophagy genes from the ATG family, such as *beclin1* and *atg5*, are overexpressed in CD34^+^ cells from chronic-phase CML patients [139] and could be repressed by miR-30a, whose expression decreased after Imatinib treatment [15]. This suggests that the TKI treatment favors a protective autophagic process, arguing in favor of the blockade of this process to render the TKI treatment fully efficient. Carella et al. have thus demonstrated that the combination of TKIs with the antibiotic Clarithromycin, that induced accumulation of LC3-II without affecting mTOR or AKT pathways, shows remarkable responses in high-risk and advanced CML-patients [30]. Spautin-1, an autophagy inhibitor which acts on Beclin-1 expression after inactivation of VSP34-AKT complexes, was shown to enhance Imatinib-induced primary CML cells apoptosis [31]. BCR-ABL was reported to control autophagy through PI3K/AKT/FOXO4 signaling, upregulating ATF5 expression that, in turn, stimulates *mTOR* transcription [140]. In this context, the PI3K/AKT/FOXO4/mTOR pathway was suggested as a druggable axis for TKI-resistant CML patients [140]. The mTOR pathway has been targeted in the clinical trials by combining Imatinib with Everolimus (NCT00093639) and Temsorilimus (NCT00101088) respectively. The PI3K/mTOR inhibitor BEZ235 used as a monotherapy, or in combination with Chloroquine, induced apoptosis in TKI-unresponsive primary CML patients’ cells [55].

Interestingly, other signaling pathways involved in the regulation of CML-LSCs self-renewal such as the Sonic Hedgehog pathway (Shh) [56] and BMI1 [92], were also reported to implicate autophagy modulation. Pharmacological inhibition of the Shh pathway by the Smo antagonist Vismodegib (GDC-0449) was associated with a markedly induced autophagy of CML cells whose simultaneous inhibition potently killed Imatinib-sensitive and -resistant BCR-ABL+ cells [56]. In addition, Vismodegib combined with Ponatinib, eliminated therapy-resistant T315I BCR-ABL cells [32]. The other Shh inhibitor PF04449913 associated with Dasatinib in a clinical trial did not exert toxicity on normal cells [141] (NCT00953758).

More recently, two second generation autophagy inhibitors, Lys05 a highly potent lysosometric agent and PIK-III a selective inhibitor of VPS34, demonstrated synergistic effects with TKIs to reduce primary CML LSCs quiescence, compared to the first-generation autophagy inhibitor Hydroxychloroquine [33].

One can note that autophagy inhibition could have beneficial roles on other CML treatments. The plant phytoalexin Resveratrol was notably reported to induce autophagic cell death in primary CML-CD34^+^ [57]. It was also reported by our group that the PRC1 component protein BMI1, that plays a key role in LSC self-renewal, prevented a protective CCNG2-dependent autophagy mechanism, that likely represents a means to support CML progression to the acute phase. Inhibition of BMI1 by the pharmacological drug PTC-209, synergized with Imatinib to strongly decrease the clonogenic properties of CD34^+^ CML cells [92].

It is well known that limited oxygen availability in the microenvironment generates a gradient of oxygen from the blood vessel to hypoxic areas which regulates mTOR that in turn inhibits autophagy [142]. In this context, Ianniciello et al., blocked autophagy using the Vps34 inhibitor in both healthy and CML-CD34^+^ and demonstrated that only healthy cells retained their capacity to proliferate after leaving hypoxia-induced quiescence, highlighting that autophagy is required for CD34^+^ CML cells [143].

#### 3.2.5. Influence of the Microenvironment on CML Resistance

The microenvironment plays a major supportive role in hematological malignancies, during leukemogenesis, but also in the appearance of resistance and relapse. The medullary microenvironment represents a complex set of vascular, nerve, immune cells, that shape not only the structure and the plasticity of the BM but also its functions. In particular, interactions of HSCs with the BM niche are fundamental to both maintain the stem cell reservoir and to produce cells for all blood lineages after asymmetric division [144]. Similarly, preservation of quiescent CML-LSCs by the BMM is essential [145] and alterations in the microenvironmental regulation by leukemic cells can play a part in the modification of the CML-LSCs response to TKIs [146].

The therapeutic efficacy of interferon-α (IFN-α) treatment was early explained by the normalization of the interactions between CML progenitors and their BMM [147] in part after restoration of a β1 integrin-mediated inhibition of hematopoietic progenitor proliferation by BM [148]. IFN-α was then clinically tested in combination with Imatinib in a phase II clinical trial (NCT00045422).

The homing of BCR-ABL+ LSCs/progenitors to recipient marrow requires numerous physical interactions. CD44 expression was reported to be increased in BCR-ABL+ stem/progenitor cells, and by engaging functional E-selectin ligands, to improve homing and engraftment of leukemic cells [149]. Furthermore, destruction of selectin ligands by neuraminidase on leukemic progenitors reduced their engraftment [150].

Wnt/β-catenin signaling from the BMM was also shown to contribute to the preservation of CML-LSCs after TKI treatment [151]. Because secretion of Wnt ligands requires their modification by the O-acyl transferase Porcupine (PORCN), the effects of the selective PORCN inhibitor WNT974 were investigated. WNT974 efficiently blocked the Wnt pathway in primary human CML cells and in association with Nilotinib, strongly decreased the colony-forming potential of CML stem/progenitors [35]. The novel Wnt/β-catenin signaling inhibitor PRI-724, associated with Nilotinib synergistically killed TKI-resistant primary BC-CML cells even under leukemia/mesenchymal stromal cells (MSCs) co-culture conditions [36]. Besides, another mechanism of resistance of CML-LSCs to TKI treatment by MSCs was identified through N-cadherin receptors and Wnt-β-catenin signaling [152]. It was reported that a paracrine loop promoted self-renewal of LSCs, by stabilizing β-catenin, involving the overexpressed AXL tyrosine kinase receptor on primary CML CD34^+^ cells and its ligand Gas6 secreted by MSCs [37]. In this context, pharmacological inhibition of AXL by the small molecules XL880 or R428, in combination with Nilotinib, strongly inhibited the growth of LSCs [37].

The bone morphogenetic protein (BMP) pathway represents another level of control of the BMM on LSCs with an over production of BMP2 and BMP4 that contributes to the dysregulation of intracellular BMP signaling in primary CP-CML samples [153]. In contrast to patients in complete cytogenetic remission, LSCs and progenitors from TKI-resistant patients displayed higher levels of BMPR1b expression and the mesenchymal cells of TKI-resistant patients increased BMP4 production, depicting a BMP autocrine loop that could account for the resistance to TKI treatment [154]. These results suggest that new pharmacological molecules against the BMP pathway could target CML-LSCs in their niche.

The CXCR4/CXCL12 axis provides survival-enhancing traits to myeloid progenitor cells and was suspected of playing a similar function in CML [155]. A high level expression of CXCR4 and of CXCL12 was notably reported in CML cells and mesenchymal stromal cells respectively [34]. Moreover, Imatinib or Nilotinib treatments of BCR-ABL+ cells induced an increased surface expression of CXCR4 [38]. Disrupting the CXCR4/CXCL12 axis by the selective CXCR4 antagonists Plerifaxor (ADM3100) [34,38,156] or BTK140 [40] lead to increase of leukemic cells’ sensitivity to TKIs. It was recently reported that CML-LSCs express the cytokine-targeting surface enzyme dipeptidylpeptidase-IV (DPPIV/CD26) that can disrupt SDF-1/CXCR4 interaction accounting for the extramedullary spread of CML-LSCs [157]. However, the specific CD26 inhibitor Vildagliptin in combination with Nilotinib did not reveal any significant cooperative effect on engraftment of CML cells in NSG mice [158].

Paracrine communication within the BMM is also crucial. For instance, BCR-ABL induced interleukin-6 (IL6) expression by BM stromal cells that in turn sustains CML development in engrafted mice [159]. This IL6 production can be the result of reprogrammed stromal cells in response to leukemic cells [160] and is suspected to decrease the self-renewal of normal progenitors. Welner et al. demonstrated that a specific IL6-blocking antibody of protected human CD34^+^ cells when exposed to CML cells and that this approach might be an effective therapy in drug-resistant CML [161].

In line with this observation, Kuepper et al. reported that IL6 could be a trigger of a JAK1-STA3 signaling pathway activation leading to the persistence of CML-LSCs; in this context, the use of JAK1 specific inhibitors (Filgotinib and Itacitinib) in combination with BCR-ABL inhibition induced apoptosis in quiescent CML-LSCs [41]. Other recent studies performed on isolated BM-derived mesenchymal stem cells (MSCs) from CML patients, showed that interleukin-7 (IL7) is highly secreted by MSCs from Imatinib-resistant patients compared to responsive patients, and that IL7 elicited Imatinib and Nilotinib resistance via a BCR-ABL independent activation of JAK1/STAT5 signaling. This opens the way for treatments combining TKIs with IL7/JAK1/STAT5 inhibitors to manage CML [162]. In this context, direct or indirect inhibition of STAT5 has been a subject of great interest. The flavonoid-like chemical compound Wogonin potentiated the inhibitory effect of Imatinib on leukemia development by suppressing STAT5 pathway in primary CML CD34^+^ cells [42]. Prost et al. used peroxysome proliferator-activated receptor-γ (PPARγ) agonists from the glitazone family and observed a marked disappearance of the CML-LSC pool after a decrease of STAT5 expression. Importantly, three patients receiving pioglitazone achieved sustained CMR, for up to 4.7 years [163]; NCT02767063). Two other phase II clinical trials have evaluated the efficiency of pioglitazone with Imatinib (NCT02687425, NCT02730195). Another study specified that pioglitazone could be combined with any of the first-, second- or third-generation TKIs to be effective against progenitor cells from both early and advanced stages CML [164].

The BMM presents a complex gradient of oxygen distribution, that differentially impacts both HSCs and LSCs [165]. LSCs are well adapted to this environment and can balance between self-renewal in low oxygen zones near the endosteal niche or engage in differentiation close to the vascular niches. The hypoxia-inducible transcription factor HIF1α that triggers an adaptive response to low oxygen concentration is required for the maintenance and survival of CML-LSCs [166]. Furthermore, physiologic hypoxia promotes maintenance of CML-LSCs independently of BCR-ABL activity [167], suggesting that targeting HIF1α in combination with a TKI could lead to complete eradication of LSCs.

#### 3.2.6. Functional Cross-Talks between the Microenvironment and Immunotherapy in CML

CML development is associated with an alteration of immune responses [168] and a better knowledge in the immunological composition of the CML BMM led to the design of a novel risk stratification model predicting patient’s response to TKI [169]. High-risk CML patients were shown to have an increased number of regulatory T cells (Treg) [170], and to highly express myeloid-derived suppressor cell (MDSC)-associated arginase 1 that can inhibit T-cell function [171]. Furthermore, as CD34^+^/CD38^−^ human CML cells express high levels of the CD25/IL2 receptor, inhibition of IL2 synthesis by cyclosporine in combination with Dasatinib was tested in a phase I clinical trial with the hope to eliminate CD25+ LSCs (NCT01426334).

On the other hand, CD34^+^ LSCs from CML patients were shown to express programmed death receptor ligand 1 (PD-L1) at diagnosis [171] whose expression was further upregulated in response to IFN-γ which could represent an interesting opportunity for T-cell immunotherapy of CML [172]. This is why some recent clinical trials are evaluating the benefit to block the PD-1/PD-L1 interaction by anti-PD-L1 antibodies on CML patients, either in combination with pioglitazone (Avelumab, NCT02767063) or Imatinib, Nilotinib or Dasatinib (Pembrolizumab, NCT03516279).

As for numerous cancer types, expression of MHC-II antigens was shown to be decreased on CML cells [58] which can participate in an immune evasion of CML cells. Tarafdar et al. have demonstrated that IFN-γ treatment of CML stem/progenitor cells induced the re-expression of MHC-II antigens, that were down-regulated by JAK1/2-mediated signals from either CML cells and/or by the CML-modified microenvironment. This suggests that associating IFN-γ with the JAK1/2 inhibitor Ruxolitinib (RUX) could favor potential immunomodulatory-based therapies [58].

Three clinical trials associating RUX and TKIs are currently in recruiting phase (NCT03654768, NCT01751425, NCT01914484) and one is terminated with encouraging results ([173], NCT01702064).

Before the use of TKIs, IFN-α treatment was the first-line therapy for CML, with some evidences of associated-immune activation particularly at the level of NK-cell cytotoxicity [174,175]. Burchert et al. found that IFN-α therapy was associated with an increased expression of leukemia-associated antigen proteinase-3 suggesting that the induction of proteinase-3-specific CTLs may contribute to sustained remission after Imatinib discontinuation [176].

Long time after being replaced by TKIs, IFN-α aroused renewed interest in CML therapy as witnessed by numerous clinical trials combining this cytokine with TKIs that show promising results [177,178]: Imatinib/IFN-α (NCT00045422; NCT00573378), Nilotinib/IFN-α (NCT01657604; NCT00573378), Dasatinib/IFN-α NCT01725204) and Bosutinib/IFN-α (NCT03831776).

#### 3.2.7. Tumor Microenvironment Impacts CML Cell’s Metabolism

LSCs from hematologic disorders establish narrow links with their stroma as a way to regulate their metabolism and redox state which are both crucial for leukemogenesis as described previously [179]. CML cells in their stromal microenvironment express specific metabolic features that represent opportunities for new treatment approaches, some of which are already being tested in clinical trials.

Earlier, it was reported that CML patients either sensitive or resistant to Imatinib showed heterogenous metabolic responses compared to their normal counterparts [180]. More recently, using liquid chromatography spectrometric metabolite profiling coupled with a multivariate statistical method, Karlikova et al. identified modifications of the metabolome of CML patients treated with TKIs, highlighting major changes in glycolysis, the citric acid cycle and amino acid metabolism [181]. Furthermore, they observed differences in the levels of amino acids and acylcarnitines between Imatinib-responder and non-responder CML patients [181]. Hattori et al. reported, in human and mouse CML models, that progression of the disease depended on a highly activated and functional expression of BCAT1, a cytosolic aminotransferase for branched-chain amino acids (BCAAs) [182]. Interestingly, eradication of LSCs has been demonstrated after targeting the amino acid metabolism in AML [183] but this LSC metabolic vulnerability has yet to be demonstrated in CML.

Besides AA metabolism, glycolysis, citric acid cycle and oxidative phosphorylation constitute numerous alternative therapies for CML. Indeed, it was shown that primary CD34^+^ CML cells depend on upregulated metabolism for their survival as demonstrated by stable isotope-assisted metabolomics and functional assays, and that the combination of Imatinib with the antibiotic tigecycline, that inhibits mitochondrial protein translation, selectively eliminates CML-LSCs [43]. SIRT1, as mentioned earlier, contributes to CML-LSCs maintenance and TKI resistance. Abraham et al. explained, by using the PGC-1α inhibitor SR18292, that the transcriptional coactivator and SIRT1-substrate, PGC-1α mediated these biological aspects by increasing oxidative phosphorylation [14]. In the same study, SR18292 strongly reduced oxygen consumption rate (OCR) on CML CD34^+^ without affecting extracellular acidification rate (ECAR) and combination with Nilotinib highly increased apoptosis and reduced clonogenicity of the leukemic cells [14].

Autophagy can also be connected to metabolism in CML cells. Another antibiotic, Ivermectin, well known for inducing autophagy by interfering with the Akt/mTOR pathway in other cancers [184], was found to induce caspase-dependent apoptosis of CML cells following induction of mitochondrial dysfunction and oxidative stress by respiratory complex I inhibition. This molecule, in combination either with Imatinib or Dasatinib, increased primary CML CD34^+^ cell death [44]. In addition, Karvela et al. demonstrated that pharmacological inhibition of autophagy or downregulation of ATG7, induced a decrease in glycolysis, an increase in oxidative phosphorylation and mitochondrial ROS accumulation thus highlighting ATG7 as a potent therapeutic target in CML [59].

Besides the BMM, Ye et al. reported that a subpopulation of LSCs expressing CD36 fatty acid transporter can use gonadal adipose tissue (GAT) as an extra-medullary niche to fuel their metabolism and evade chemotherapy [185].

Interestingly, BCL2 inhibition was demonstrated to reduce oxidative phosphorylation and selectively eradicate in AML-LSCs [186]. The previously described efficacy of BCL2 inhibition [25] in CML could be partially explained by this mechanism.

#### 3.2.8. Targeting Signaling Pathways Connected to Key Leukemic Stem Cells Features

Self-renewal of LSCs is regulated by multiple pathways such as the Wnt, Shh, BMI1 pathways and is highly dependent on the tumor microenvironment as previously described. Blocking this fundamental stem cell function through inhibition of these pathways is an obvious strategy. There has been an accumulation of studies showing a strong role of Wnt/β-catenin in blastic transformation [187], in survival of BCR-ABL leukemic cells [188] and in self-renewal of CML-LSCs [189]. Interestingly, CML-LSCs were shown to express CD27 that responds to the tumor necrosis factor (TNF) family ligand CD70 to increase expression of Wnt target genes [190]. Furthermore, because expression of CD70 is restricted to activated lymphocytes and dendritic cells its engagement could account for an adaptive immune response to leukemia progression [190]. More recently, Riether et al. demonstrated that TKIs induced the expression of CD70 in LSCs triggering CD27 signaling in a cell-autonomous and/or paracrine manner. The blockade of CD70 by a specific antibody combined with Imatinib effectively eliminated human CD34^+^ CML progenitor/stem cells [45]. Despite all these possibilities, the only pharmacological approach tested on primary CML CD34^+^ cells are the selective PORCN inhibitor, WNT974 described above [35].

Smoothened (Smo), a component of the Hedgehog (Shh) pathway involved in LSCs self-renewal, was considered as a promising target to eliminate LSCs despite its important role in the maintenance of normal HSCs [191]. Indeed, two different studies demonstrated that targeting the Shh pathway may provide some benefits. It was first observed that overexpression of miR-326 downregulated Smo to induce an elevated rate of apoptosis in CML CD34^+^ cells [60]. In addition, inhibition of Shh by the Smo antagonist LDE225 (Sonidegib) in combination with Imatinib, reduced the self-renewal capacity of CD34^+^ CP-CML and their engraftment in NSG mice, without affecting HSCs [46]. Sonidegib was also combined with Nilotinib in the treatment of chronic or accelerated phase in patients who developed resistance to first-line therapy (NCT01456676). Besides, the specific targeting of Smo has been evaluated in clinical trials in combination with Dasatinib (BMS-833923; NCT01218477 and NCT01357655) (PF04449913; NCT00953758).

BCR-ABL controls many signaling pathways some of which are essential for resistance to TKI-induced apoptosis: PI3K/AKT [192], JAK/STAT [193,194], or Ras/MEK/ERK [195] and represent therefore good secondary targetable candidates to reach a cure for CML [196].

AKT can inhibit FOXO-mediated transcription of genes required for apoptosis such as Bim [197]. More precisely, Naka et al. describe that FOXO3a plays an essential role in the maintenance of CML-LSCs in a CML-like myeloproliferative disease mouse model [47] and that TGF-β activates AKT in LSCs by controlling FOXO3a localization [47]. Furthermore, the association of the TGF-β inhibitors, Ly364947 with Imatinib [47] or EW-7197 with Imatinib, Dasatinib or Ponatinib [11] was efficient to respectively decrease the maintenance of LSCs and to eliminate CML leukemia-initiating cells.

As discussed above in the autophagy section, the clinically well tolerated mTOR inhibitor RAD001 was used to block Imatinib-induced AKT activation that mediates survival during the early phase of Imatinib resistance [198].

Janus kinase 2 (JAK2) has recently emerged as an attractive target to improve TKI treatment in CML as demonstrated *in-vitro* [199]. Moreover, the JAK2 inhibitor BMS-911543 selectively targets CML-LSCs without affecting healthy progenitor/stem cells and its association with Dasatinib effectively eliminated primary TKI-insensitive CML-LSCs [48]. The other JAK2 inhibitor RUX was successfully combined with Nilotinib to lead to a strong reduction in primitive quiescent CML stem cells [50].

The Ras/MEK/ERK pathway can be targeted at different levels and many molecules have been tested in combination with TKIs. Farnesyl transferase inhibitors such as BMS-214662 (that inactivate Ras by inhibiting its recruitment to the plasma membrane) have been associated with Imatinib in a phase I clinical trial (Zarnestra; NCT00040105 and Lonafarnib; NCT00047502). MEK could also be a potential therapeutic target, as its specific MEK inhibitor PD184352, when combined with BMS-214662 increased apoptosis in TKI-unresponsive CD34^+^ CML cells [61]. Lonafernib that inhibits Raf was evaluated in a phase I clinical trial (NCT00047502). Imatinib was also tested with the dual Raf/VEGFR inhibitor Sorafenib in a phase II clinical trial (NCT00661180) and with the VEGFR inhibitor Vatalanib in a phase I/II study (NCT00088231). Grb2 is a potent activator of ERK1 and ERK2 in Ph+ leukemic cells [200], and the use of BP1001, a liposome-packaged Grb2 antisense oligonucleotide is well tolerated in CML patients [201] and is evaluated in combination with Dasatinib in a phase I/II clinical trial (NCT02923986).

Niclosamide, an FDA-approved anthelmintic drug, inhibits the overexpressed ERK/MNK1/eIF4E pathway which makes BP-CML more sensitive to Dasatinib [49]. The MNK inhibitor CGP57380 was as efficient as Dasatinib to impair LSCs function [62]. Activation of β-catenin was linked to CML drug resistance through BCR-ABL impaired binding to GSK3β (glycogen synthase kinase 3β). The specific GSK3 inhibitor SB216763, associated with Imatinib led to an almost complete suppression of primary CML progenitors/stem cells but failed to do so when combined with Dasatinib [202].

The dysregulation of the serine/threonine Aurora kinases (A, B and C) activity is well known to induce chromosome instability and aberrant mitosis in hematologic malignancies [203]. Targeting this pathway in CML, and particularly in TKI-resistant patients, emerged quite early. In this line, the pan-Aurora Kinase inhibitor MK-0457 (VX-680), has been investigated for the treatment of resistant CML patients harboring the BCR-ABL mutation T315I with encouraging results at a dose that did not induce side effects [204] while 44% of the patients had hematological responses [205], and cytogenetic responses were observed in 14% of patients in a phase II study ([206]; NCT00111683). A phase I study also evaluated the combination of Dasatinib and MK-0457 on CML patients (NCT00500006).

On the other hand, the arachidonic acid (AA)-leukotriene (LT) pathway has been demonstrated to be implicated in the LSCs survival. *Alox15,* that encodes for the 15-lipoxygenase (15-LO), has been shown to play an essential role in the functional regulation of CML-LSCs and specific inhibition of 15-LO by PD146176 leads to human CML CD34^+^ death with a synergy when combined either with imatinib or nilotinib [51]. Among the products of 15-LO, LTB4 can stimulate BLT2 receptor whose expression is significantly increased in CML CD34^+^ stem/progenitors, and inhibition of this pathway by the BLT2-specific inhibitor LY255283 induced apoptosis and inhibited self-renewal capacity of CD34^+^ cells from TKI-resistant BP-CML patients [63].

## 4. Conclusions

CML has benefited from three generations of TKIs, which have transformed the leukemia into a perfectly controlled disease but for which interruption of the monotherapy is often associated with relapse due to persisting LSCs.

Numerous studies have identified molecular and functional dysregulations in LSCs as well as specific relationships between CML cells and their medullary tissue environment, which can also be involved in the emergence of TKI resistance. The development of molecules targeting these different situations has frequently demonstrated that it was possible to improve the sensitivity of LSCs to TKIs supporting the development of combinatorial treatments. Association of TKI with other drugs recently emerged as stronger solutions to eliminate LSCs that in some cases are being investigated in clinical trials.

Thus, in the past two decades, studies have first shown that it may be interesting to target epigenetic and apoptotic functions. Numerous ex-vivo studies have also described the autophagic survival process as a potential target leading to LSCs eradication that should soon be tested in clinical trials. The most recent insights concern metabolic differences between LSCs and HSCs, like mitochondrial bioenergetics and indirect or direct targeting of the mitochondrial respiratory chain have already been tested on primary CML cells and need to be evaluated in clinical investigations.

Other new strategies aimed at targeting the tumor microenvironment, mostly inspired from ex-vivo experiments, have already made their way to clinical trials. The emerging success of immunotherapies targeting immune checkpoints in other cancers will probably be an additional option to improve the efficacy of TKIs in CML.

Recent technological advances are allowing new studies deciphering the heterogeneity between cancer cells and the establishment of predictive models for TKIs response. Current developments in single-cell transcriptomic analysis identified a blast-crisis specific stem cell population resistant to a TKI treatment [207].

NGS is now the gold standard method to detect early occurrences of kinase domain mutations, that helps to predict relapses [208,209].

In parallel, Single Cell Analysis is a promising emerging approach to identify genome-scale molecular information at the single cell level. By combining transcriptomic and proteomic analyses, Abraham et al. showed, at the CML patient level the therapeutic potential of RG7112/7388 (HDM2 inhibitors) and CPI-203/0610 (BET inhibitors) combination to target p53 and c-MYC respectively in LSC maintenance [64].

With up-to-date technologies, the fundamental knowledge about the mechanisms which govern LSCs response to TKIs according to individuals will improve. We can then expect in the near future that multi-drug regimens, developed around TKIs, can be proposed to tackle the different molecular or cellular pathways associated with TKI resistance that have been described here, aiming ultimately at the development of personalized treatments.

## Figures and Tables

**Table 1:** Chronic Myeloid Leukemia (CML) Treatments with Ex-Vivo Evidences of Effectiveness either in Combination with tyrosine kinase inhibitor (TKIs) or Alone.

Scheme Number	Treatment 1	Treatment 2	Pathway	References
1	Ponatinib	Asciminib	BCR-ABL1 allosteric inhibitor	[7]
2	Imatinib Dasatinib Nilotinib	GSK343	EZH2 inhibitor	[8]
3	Imatinib	LBH589	HDAC inhibitor	[9]
4	Nilotinib	LBH589	HDAC inhibitor	[10]
5	Imatinib	LAQ824	HDAC inhibitor	[11]
6	Imatinib	Hydralazine and Magnesium Valporate	HDAC inhibitors	[12]
7	Imatinib	Tenovin-6	SIRT1 inhibitor	[13]
8	Nilotinib	TV39OH	SIRT1 inhibitor	[14]
9	Imatinib	miR30a inhibition	Genetic repressor/activator	[15]
10	Imatinib	miR-202 overexpression	Inhibition of hexokinase 2 (HK2) and glycolysis	[16]
11	Imatinib	miR-486	Genetic repressor/activator	[17]
12	Imatinib	Ovatodiolide	miR-155 upregulation/PI3K/mTOR inhibition	[18]
13	Imatinib	RI-BPI	BCL6 inhibitor	[19]
14	Imatinib	FX1	BCL6 inhibitor	[20]
15	Imatinib	A-1210477	MCL-1 inhibitor	[20]
16	Dasatinib	Sabutoclax	Pan-BCL2 inhibitor	[21]
17	Imatinib	ABT-737	BCL2 and Bcl-xL inhibitor	[22,23]
18	Nilotinib	Nutlin3a ABT-737	MDM2 inhibitor BCL2 and Bcl-xL inhibitor	[24]
19	Imatinib	DS-5272	MDM2 inhibitor	[25]
20	Imatinib	ABT-199 (Venetoclax)	BCL2 inhibitor	[26]
21	Nilotinib	ABT-199 (Venetoclax)	BCL2 inhibitor	[27]
22	Imatinib Nilotinib Dasatinib	Chloroquine Bafilomycin A1	Autophagy inhibitors	[9,28]
23	Imatinib	Chloroquine Bafilomycin A1	Autophagy inhibitors	[29]
24	Imatinib Nilotinib Dasatinib	Clarithromycin	Autophagy inhibitor	[30]
25	Imatinib	Spautin-1	Autophagy inhibitor	[31]
26	Ponatinib	Vismodegib (GDC-0449)	Smo antagonist Hedgehog pathway	[32]
27	Nilotinib	Lys05	Autophagy inhibitor	[33]
28	Nilotinib	PIK-III	VPS34 inhibitor Autophagy	[33]
29	Imatinib	PTC-209	BMI1 inhibitor	[34]
30	Nilotinib	WNT974	PORCN selective inhibitor Wnt pathway	[35]
31	Nilotinib	PRI-724	CBP inhibitor Wnt/β-catenin inhibition	[36]
32	Nilotinib	XL880 R428	AXL inhibitors	[37]
33	Imatinib	Plerifaxor (ADM3100)	CXCR4 antagonist	[34]
34	Imatinib Nilotinib	Plerifaxor (ADM3100)	CXCR4 antagonist	[38]
35	Nilotinib	Plerifaxor (ADM3100)	CXCR4 antagonist	[39]
36	Imatinib	BTK140	CXCR4 antagonist	[40]
37	Imatinib	Filgotinib Itacitinib	JAK1 specific inhibitors, JAK1/STAT3 pathway	[41]
38	Imatinib	Wogonin	STAT5 pathway inhibitor	[42]
39	Imatinib	Tigecycline	Mitochondrial ribosome protein translation and respiratory chain	[43]
40	Nilotinib	SR18292	PGC-1α inhibitor/SIRT1 pathway inhibition	[14]
41	Imatinib Dasatinib	Ivermectin	mTOR inhibitor	[44]
42	Imatinib	Anti-CD70	Wnt/β-catenin inhibition	[45]
43	Imatinib	Sonidegib (LDE225)	Smo antagonist Hedgehog pathway inhibition	[46]
44	Imatinib	Ly364947	TGF-βRI inhibitor TGFβ/Activin/NODAL pathway	[47]
45	Imatinib Dasatinib Ponatinib	EW-7197	TGF-β signaling inhibitor	[11]
46	Dasatinib	BMS-911543	JAK2 inhibitor	[48]
47	Dasatinib	Niclosamide	ERK/MNK1/eIF4E pathway inhibition	[49]
48	Imatinib	SB216763	GSK-3 specific inhibitor	[50]
49	Imatinib Nilotinib	PD146176	15-LO inhibition/ arachidonic acid -leukotriene pathway inhibition	[51]
50	none	EZH2 CRISPR/Cas9 invalidation	EZH2 inhibition	[52]
51	none	Pracinostat	HDAC inhibitor	[53]
52	none	PJ-68	Methyltransferase inhibitor (PRMT5) Wnt/β-catenin pathway inhibition	[54]
53	none	Dactolisib (BEZ235) Chloroquine	PI3K/mTOR inhibitor Autophagy inhibitor	[55]
54	none	Vismodegib (GDC-0449)	Smo antagonistHH pathway inhibition	[56]
55	none	Resveratrol	Autophagic cell death induction	[57]
56	none	IFN-α Ruxolitinib	MHC II increased expression JAK1/2 inhibitor	[58]
57	none	shATG7	Mitochondrial ROS and oxidative phosphorylation increase Autophagy inhibition	[59]
58	none	miR-326 overexpression	Downregulation of SmoHH pathway inhibition	[60]
59	none	PD184352 BMS-21462	MEK specific inhibitor Farnesyl transferase inhibitor	[61]
60	none	CGP57380	MNK1 inhibitor ERK/MNK1/eIF4E pathway inhibition	[62]
61	none	LY255283	BLT2 inhibitor/arachidonic acid -leukotriene pathway inhibition	[63]
62	none	RG7112/7388 CPI-203/0610	HMD2 inhibitors (p53 inhibition) BET inhibitors (c-Myc inhibition)	[64]

**Table 2:** Clinical Trials Involving CML Patients Treated either with Investigational Molecules in Combination with TKIs or Alone.

Scheme Number	Treatment 1	Treatment 2	Pathway	Investigation	Phase	Identifier	First Posted	Status
1	Imatinib Nilotinib Dasatinib	Asciminib	BCR-ABL1 allosteric inhibitor	Frontline combination in CP- CML	II	NCT03906292	2019	Recruiting
2	none	Asciminib	BCR-ABL1 allosteric inhibitor	Efficacy of ABL001 versus Bosutinib in CP-CML patients previously treated with TKIs	III	NCT03106779	2017	Recruiting
3	Dasatinib	Asciminib	BCR-ABL1 allosteric inhibitor	Combination in CML in lymphoid blast crisis	I	NCT03595917	2018	Recruiting
4	Imatinib	Asciminib	BCR-ABL1 allosteric inhibitor	Efficacy and safety of combination in patients with CP-CML	II	NCT03578367	2018	Recruiting
5	Imatinib Nilotinib Dasatinib	Asciminib	BCR-ABL1 allosteric inhibitor	Oral ABL001 in CML Patients	I	NCT02081378	2014	Recruiting
6	Imatinib	Cytarabine	DNA synthesis inhibitor	Combination in patients with CML	II	NCT00022490	2003	Terminated
7	Imatinib	Cytarabine	DNA synthesis inhibitor	Combination in patients with CML	I/II	NCT00015834	2003	Completed
8	Dasatinib	SAHA (Vorinostat)	HDAC inhibitor	Combination in treating patients with accelerated phase or BP- CML	I	NCT00816283	2009	Completed
9	Imatinib	Panobinostat (LBH589)	HDAC inhibitor	Safety and tolerability of LBH589 combined with imatinib in CML patients in MCR	I	NCT00686218	2008	Completed
10	Imatinib	Decitabine	DNA methyltransferase inhibitor	Combination in patients with CML	II	NCT00054431	2003	Completed
11	Dasatinib	Venetoclax	BCL2 inhibitor	Combination in treating patients with BCR-ABL1 positive early chronic phase	II	NCT02689440	2016	Recruiting
12	Ponatinib	Venetoclax Dexamethasone	BCL2 inhibitor Anti-inflammatory	Triple combination in BCR-ABL positive relapsed CML	I/II	NCT03576547	2018	Recruiting
13	Imatinib	Oblimersen	bcl-2 antisense oligodeoxynucleotide	Oblimersen and Imatinib in treating patients with CML	II	NCT00049192	2003	Completed
14	Imatinib	Hydroxychloroquine	Autophagy inhibitor	Effectiveness of combination on BCR/ABL levels in CML patients in MCR	II	NCT01227135	2010	Unknown
15	Imatinib	Everolimus (RAD001)	mTOR inhibitor	Combination in patients in CP-CML who are not in CCR after previous Imatinib	I/II	NCT00093639	2004	Completed
16	Imatinib	Temsirolimus	mTOR inhibitor	Temsirolimus and Imatinib in treating patients with CML	I	NCT00101088	2005	Terminated
17	Dasatinib	PF04449913 (Glasdegib)	Smo antagonist HH inhibition	Study of PF-04449913 in select hematologic malignancies	I	NCT00953758	2009	Completed
18	Imatinib	Interferon-α	Immunomodulatory effect	INF-α and Imatinib in CML patients	II	NCT00045422	2003	Completed
19	Imatinib	Pioglitazone	PPARγ agonist STAT5 inhibition	Combination in patients with relapsed CML	II	NCT02767063	2016	Terminated
20	Imatinib	Pioglitazone	PPARγ agonist STAT5 inhibition	Efficiency of combination to treat CML	II	NCT02687425	2016	Unknown
21	TKIs	Pioglitazone	PPARγ agonist STAT5 inhibition	Pioglitazone and TKI in patients with relapsed CML	II	NCT02730195	2019	Terminated
22	Dasatinib	Cyclosporine	IL-2 inhibitor	Combination in patients with CML refractory or intolerant to imatinib	I	NCT01426334	2011	Terminated
23	Pioglitazone	Avelumab (Anti-PD-L1)	PPARγ agonist STAT5 inhibition PD-1/PD-L1 inhibition	Therapies in combination or sequentially with TKIs in CP-CML patients in CCR (ACTIW)	I/II	NCT02767063	2016	Recruiting
24	Imatinib Nilotinib Dasatinib	Pembrolizumab (Anti-PD-1)	PD-1/PD-L1 inhibition	Pembrolizumab and TKIs in CML patients with persistently detectable MRD	II	NCT03516279	2018	Recruiting
25	Dasatinib Nilotinib	Ruxolitinib	JAK1/2 selective inhibitor	Ruxolitinib phosphate and Dasatinib or Nilotinib in treating CML patients	II	NCT03654768	2018	Recruiting
26	Nilotinib	Ruxolitinib	JAK1/2 selective inhibitor	Ruxolitinib in treating participants with CML with MRD after TKIs	I/II	NCT01751425	2012	Active, not recruiting
27	Nilotinib	Ruxolitinib	JAK1/2 selective inhibitor	Nilotinib/Ruxolitinb therapy for TKI resistant Ph-Leukemia	I//II	NCT01914484	2013	Unknown
28	Nilotinib	Ruxolitinib	JAK1/2 selective inhibitor	Combination in CP-CML Patients	I	NCT01702064	2012	Completed
29	Nilotinib	Peginterferon α2b	Immunomodulatory effect	Evaluation of TKI and INF-α	III	NCT01657604	2012	Active, not recruiting
30	Imatinib Nilotinib	Peginterferon α2b	Immunomodulatory effect	Imatinib or Nilotinib with Pegylated Interferon-α2b in CML	II	NCT00573378	2007	Withdrawn
31	Dasatinib	Peginterferon α2b	Immunomodulatory effect	Safety and efficacy of combination in newly diagnosed CML (NordCML007)	II	NCT01725204	2012	Completed
32	Bosutinib	Ropeginterferon	Immunomodulatory effect	Long-acting low dose Ropeginterferon for CML treated with Bosutinib from diagnosis	II	NCT03831776	2019	Recruiting
33	Nilotinib	Sonidegib (LDE225)	Smo antagonist HH inhibition	Combination in CML patients who developed resistance to prior therapy	I	NCT01456676	2011	Completed
34	Dasatinib	BMS-833923	Smo antagonist HH inhibition	Combination therapy in CML	I/II	NCT01218477	2010	Completed
35	Dasatinib	BMS833923	Smo antagonist HH inhibition	Dasatinib combo with Smo antagonist	II	NCT01357655	2011	Terminated
36	Dasatinib	PF04449913	Smo antagonist HH inhibition	Study in select hematologic malignancies	I	NCT00953758	2009	Completed
37	Imatinib	Zarnestra	Farnesyltransferase inhibitor	Zarnestra and Gleevec in CP- CML	I	NCT00040105	2002	Completed
38	Imatinib	Lonafarnib	Farnesyltransferase inhibitor	Lonafarnib and Gleevec in CML	I	NCT00047502	2002	Completed
39	none	Sorafenib (BAY43-9006)	Raf/VEGFR/PDGFR inhibitor	Raf kinase inhibitor BAY 43-9006 in CML patients resistant to Gleevec	II	NCT00661180	2008	Completed
40	Imatinib	Vatalanib (PTK 787)	VEGFR inhibitor	Combination in patients with BP-CML	I/II	NCT00088231	2004	Completed
41	Dasatinib	BP1001	Grb2 inhibitor	Combination of liposomal Grb2 antisense oligonucleotide with Dasatinib in CML patients	I/II	NCT02923986	2016	Recruiting
42	none	MK0457	Aurora kinase inhibition	MK0457 in CML patients (0457-003)	I	NCT00111683	2005	Completed
43	Dasatinib	MK0457	Aurora kinase inhibition	Evaluation of efficacy and safety in patients with CML	I	NCT00500006	2007	Terminated

## Abbreviations

15-LO	15-Lipoxygenase
4E-BP1	Eukaryotic translation initiation factor 4E-binding protein 1
A-loop	Activation-loop
AA	Arachidonic acid
ABC	ATP-binding cassette
ABCB1/MCR1	Multidrug resistance protein 1/Permeability glycoprotein
ABCC1/MRP1	Multidrug resistance-associated protein 1
ABCG2/BCRP	ATP-binding cassette super-family G member 2
AKT/PKB	Protein kinase B
AML	Acute Myeloid Leukemia
AMP	Adenosine Monophosphate
AMPK	AMP-activated protein kinase
AP	Adenomatous Polyposis Coli
Ara-C	Cytarabine
ATG	Autophagy related genes
ATP	Adenosine Tri-Phosphate
AXL	Tyrosine-protein kinase receptor UFO
BCL-2	B-cell lymphoma 2
BCL6	B-cell lymphoma 6
Bcl-xL	B-cell lymphoma-extra large
BCR-ABL	Breakpoint Cluster Region-Abelson murine leukemia viral oncogene homolog 1
BET	Bromodomain Extra-Terminal motif
BH3	Bcl-2 homology domain 3
BIM	Bcl-2-like protein 11
BLT2	Leukotriene B4 receptor 2
BM	Bone Marrow
BMI1	B cell-specific Moloney murine leukemia virus integration site 1
BMM	Bone marrow microenvironment
BMP	Bone morphogenetic proteins
BP-CML	Blastic phase chronic myeloid leukemia
c-ABL	Abelson murine leukemia viral oncogene homolog 1
C/EBPβ	CCAAT/Enhancer-binding Protein beta
CBL	Casitas B-lineage Lymphoma
CCNG2	Cyclin G2
CCR	Complete Cytogenetic Remission
CHR	Complete Hematological Remission
CITED2	Cbp/p300-interacting transactivator 2
CpN	Cyclophilin
CML	Chronic Myeloid Leukemia
CQ	Chloroquine
CRK	CT10 Regulator of Kinase
CRKL	Crk-like protein
CsA	Cyclosporine
CXCL12	C-X-C motif chemokine 12
CXCR4	C-X-C chemokine receptor type 4
Cyt	Cytokines
CytR	Cytokine receptors
DNA	Deoxyribonucleic acid
DVL	Dishevelled
ECAR	Extracellular Acidification Rate
EIF4E	Eukaryotic translation initiation factor 4E
ERK	Extracellular signal-regulated kinases
ETC	Electron Transport Chain
EZH2	Enhancer Of Zeste 2 Polycomb Repressive Complex 2 Subunit
FOXO	Forkhead box protein O
GAB2	GRB2-associated-binding protein 2
GAS6	Growth arrest–specific 6
GLI1	Glioma-associated oncogene homologue 1
GRB2	Growth factor receptor-bound protein 2
GSK-3β	Glycogen Synthase Kinase 3 Beta
H3K27me3	Histone H3 trimethylase activity at lysine 27
HCQ	Hydroxychloroquine
HIF-2α	Hypoxia-inducible factor-2 alpha
HDAC	Histone deacetylase
HSC	Hematopoietic Stem Cell
IFN	Interferon
IL	Interleukin
ILR	Interleukin Receptor
JAK	Janus-family tyrosine kinase
LC3	microtubule-associated protein 1 light chain 3
LIC	Leukemia-Initiating Cells
LRP5/6	Low density lipoprotein receptor-related protein 5/6
LSC	Leukemic Stem Cell
LT	Leukotriene
LTB4	Leukotriene B4
MCL-1	induced myeloid leukemia cell differentiation protein
MCR	Major Cytogenetic Remission
MDR	Multidrug Resistance
MDM2	Mouse double minute 2 homolog
MEK	Mitogen-activated protein kinase kinase;
MHC	Major histocompability complex
miRNA	Micro ribo nucleic acid;
MNK	MAP kinase-interacting serine/threonine-protein kinase
MPT	Mitochondrial protein translation
mTOR	mechanistic Target Of Rapamycin
NFAT	Nuclear factor of activated T-cells
NSG (NOD/SCID)	Nonobese diabetic/severe combined immunodeficient
OCR	Oxygen Consumption Rate
P-loop	Phosphate-binding loop
PAK1	Serine/threonine-protein kinase 1
PD-1	Programmed cell death 1
PD-L1	Programmed death-ligand 1
PGC-1α	Peroxisome proliferator-activated receptor gamma coactivator 1-alpha
PI3K-III	Phosphoinositide 3-kinase class III
PORCN	Porcupine O-acyltransferase
PPARγ	Peroxisome proliferator-activated receptor gamma
PRC	Protein regulator of cytokinesis
PTCH	Protein patched homolog 1
RAF	Rapidly accelerated fibrosarcoma kinases
RPS6	Ribosomal protein S6
SH2/SH3	SRC Homology 2/3 Domain
SHC	SH2-contaltining collagen-related proteins
SHH	Sonic Hedgehog
SIRT1	Sirtuin 1
SMO	Smoothened
SOS	Son Of Sevenless guanine nucleotide exchange factor
STAT	Signal transducer and activator of transcription;
TCR	T cell receptor
TGF-β1	Transforming growth factor beta 1
TGF-βR	abrogated transforming growth factor beta receptor
TKI	Tyrosine Kinase Inhibitor
TNF	Tumor Necrosis Factor
Ub	Ubiquitin
ULK1	unc-51 like autophagy activating kinase 1
VEGF	Vascular endothelial growth factor
VEGFR	Vascular endothelial growth factor receptor
WNT	Wingless-related integration site
